# Effect of Growth Medium pH of *Aeropyrum pernix* on Structural Properties and Fluidity of Archaeosomes

**DOI:** 10.1155/2012/285152

**Published:** 2012-06-13

**Authors:** Ajda Ota, Dejan Gmajner, Marjeta Šentjurc, Nataša Poklar Ulrih

**Affiliations:** ^1^Department of Food Science and Technology, Biotechnical Faculty, University of Ljubljana, Jamnikarjeva 101, 1000 Ljubljana, Slovenia; ^2^EPR Center, Institute Jožef Stefan, Jamova 39, 1000 Ljubljana, Slovenia; ^3^Centre of Excellence for Integrated Approaches in Chemistry and Biology of Proteins (CipKeBiP), Jamova 39, 1000 Ljubljana, Slovenia

## Abstract

The influence of pH (6.0; 7.0; 8.0) of the growth medium of *Aeropyrum pernix* K1 on the structural organization and fluidity of archaeosomes prepared from a polar-lipid methanol fraction (PLMF) was investigated using fluorescence anisotropy and electron paramagnetic resonance (EPR) spectroscopy. Fluorescence anisotropy of the lipophilic fluorofore 1,6-diphenyl-1,3,5-hexatriene and empirical correlation time of the spin probe methylester of 5-doxylpalmitate revealed gradual changes with increasing temperature for the pH. A similar effect has been observed by using the trimethylammonium-6-diphenyl-1,3,5-hexatriene, although the temperature changes were much smaller. As the fluorescence steady-state anisotropy and the empirical correlation time obtained directly from the EPR spectra alone did not provide detailed structural information, the EPR spectra were analysed by computer simulation. This analysis showed that the archaeosome membranes are heterogeneous and composed of several regions with different modes of spin-probe motion at temperatures below 70°C. At higher temperatures, these membranes become more homogeneous and can be described by only one spectral component. Both methods indicate that the pH of the growth medium of *A. pernix* does not significantly influence its average membrane fluidity. These results are in accordance with TLC analysis of isolated lipids, which show no significant differences between PLMF isolated from *A. pernix* grown in medium with different pH.

## 1. Introduction

Archaea are the third domain of living organisms, and they have cell structures and components that are markedly different from those of bacteria and eukaryotes. The glycerol ether lipids are the main feature that distinguishes the members of archaea from bacteria and eukarya [1]. In contrast to bacteria and eukarya, where the acyl chains of the membrane phospholipids are ester-linked to the *sn*-glycerol-3-phosphate scaffold, the backbone of archaeal lipids is composed of *sn*-glycerol-1-phosphate, with isoprenoid groups connected *via* ether linkages [2–7]. 

**Figure 1:**
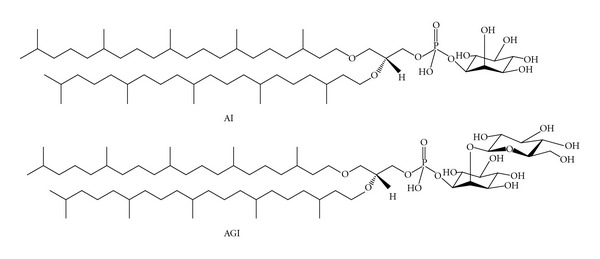
Structural formulas of 2,3-di-*O*-sesterpanyl-*sn*-glycerol-1-phospho-myo-inositol (C_25,25_-archetidylinositol) (top: AI) and 2,3-di-*O*-sesterpanyl-*sn*-glycerol-1-phospho-1′-(2′*O*-*α*-D-glucosyl)-myo-inositol (C_25,25_-archetidyl(glucosyl)inositol) (bottom: AGI).

*Aeropyrum pernix* K1 was the first absolutely aerobic, hyperthermophilic archaeon that was isolated from a costal solfataric thermal vent in Japan [8]. The polar lipids of *A. pernix* K1 consist solely of C_25,25_-archaeol (2,3-di-sesterpanyl-*sn*-glycerol), with C_25,25_-archetidyl(glucosyl)inositol (AGI) accounting for 91 mol%, and C_25,25_-archetidylinositol (AI) accounting for the remaining 9 mol% (Figure 1). Membranes composed of such C_25,25_ diether lipids have 20% greater thickness than those composed of tetraether C_20,20_ archaeal-based lipids [9]. 

Over the last five years, we have investigated the influence of some environmental factors on the structural properties of the membrane of *A. pernix in vivo* using electron paramagnetic resonance (EPR) and fluorescence emission spectrometry [10]. These studies included the influence of pH and temperature on the physiochemical properties of bilayer archaeosomes prepared from a polar-lipid methanol fraction (PLMF) isolated from *A. pernix* cells grown at 92°C at pH 7.0, of mixed liposomes prepared from mixtures of this PLMF and 1,2-dipalmitoyl-*sn*-glycero-3-phosphoholine (DPPC) at different ratios [11–13]. The major conclusion based on our differential scanning calorimetry (DSC) was that the archaeosomes do not show gel to liquid crystalline phase transition in the temperature range from 0 to 100°C [11]. 

Through these investigations of *A. pernix in vivo*, we have shown that the growth medium pH influences the initial growth rate and cell density [14]. A pH below 7.0 was less favourable than pH 8.0, and there was no growth of *A. pernix* at pH 5.0. Using the EPR and fluorescence emission measurements, changes in the distribution of the spin probes and their motional characteristic were monitored. These changes reflect the changes in the membrane domain structure with temperature, and they were different for *A. pernix* grown at pH 6.0 than at pH 7.0 and 8.0 [10]. Macalady and coworkers (2004) [15] suggested that there is a strong correlation between core-lipid composition and optimal pH of the growth medium. 

In the present study, we have extended our EPR and fluorescence emission spectrometry to investigate the influence of growth medium pH (6.0; 7.0; 8.0) on the physiochemical properties of bilayer archaeosomes prepared from this PLMF from *A. pernix*. 

## 2. Materials and Methods

### 2.1. Growth of *A. pernix* K1

*A. pernix* K1 was purchased from Japan Collection of Microorganisms (number 9820; Wako-shi, Japan). The culture medium comprised (per litre): 34.0 g marine broth 2216 (Difco Becton, Dickinson & Co., Franklin Lakes, NJ, USA), 5.0 g Trypticase Peptone (Becton, Dickinson and Company, Sparks, USA), 1.0 g yeast extract (Becton, Dickinson and Company, Sparks, USA) and 1.0 g Na_2_S_2_O_3_•5H_2_O (Sigma-Aldrich, St. Louis, USA). The buffer systems used were 20 mM MES [2-(N-morpholino)ethanesulfonic acid; Acros Organics, Geel, Belgium] for growth at pH 6.0, and 20 mM HEPES [4-(2-hydroxyethyl)-1-piperazineethanesulfonic acid; Sigma-Aldrich Chemie GmbH, Steinheim, Germany] for growth at pH 7.0 and pH 8.0. The *A. pernix *cells were grown in 800 mL growth medium in 1000 mL heavy-walled flasks, with a magnetic stirring hot plate and forced aeration (0.5 L·min^−1^) at 92°C, as described previously [14]. 

### 2.2. Isolation and Purification of Lipids

**Figure 2:**
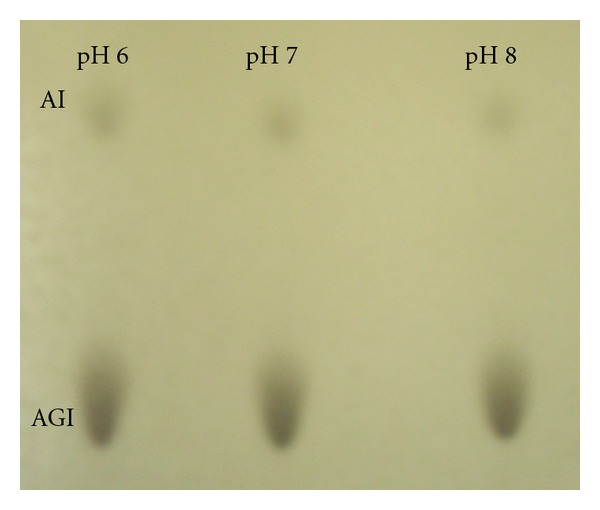
TLC results of PLMF from *A. pernix* grown at different pH as marked. AI and AGI stands for C_25,25_-archetidylinositol and C_25,25_-archetidyl(glucosyl)inositol, respectively.

The PLMF that is composed of approximately 91% AGI and 9% AI (average molecular weight of 1181.42 g·mol^−1^) was prepared from the lyophilised *A. pernix* cells as described previously [11]. The lipids were fractionated using adsorption chromatography and analysed by TLC with the chloroform/methanol/acetic acid/water (85/30/15/5) solvent. Analysis was performed by 0.04 mg of PLMF isolated from *A. pernix* grown at different pH. TLC plate was developed and sprayed with 20% H_2_SO_4_.Lipid spots were visualized by heating at 180°C for 20 minutes [9]. TLC plates were analysed using JustTLC software (Version 3.5.3. http://www.sweday.com/), where intensity ratio of the two lipid components was compared. No differences between PLMF isolated from *A*. *pernix* grown in medium with different pH were observed (Figure 2).

The methanol fraction containing the polar lipids (PMLF) was used for further analysis. This lipid solution was dried by slow evaporation under a constant flow of dry nitrogen, followed by vacuum evaporation of solvent residues. 

### 2.3. Preparation of Archaeosomes

The appropriate weights of the dried PLMF were dissolved in chloroform and transferred into glass round-bottomed flasks, where the solvent was evaporated under reduced pressure (17 mbar). The dried lipid films were then hydrated with the aqueous buffer solutions. As indicated above, the following 20 mM buffer solutions were used: MES for pH 6.0 and HEPES for pH 7.0 and 8.0. The final concentration of the lipids was 10 mg*·*mL^−1^. Multilamellar vesicles (MLVs) were prepared by vortexing the lipid suspensions for 10 min. The MLVs were further transformed into small unilamellar vesicles (SUVs) by 30 min sonication with 10 s on-off cycles at 50% amplitude with a Vibracell Ultrasonic Disintegrator VCX 750 (Sonics and Materials, Newtown, USA). To separate the debris from SUVs after sonification, the sample was centrifuged for 10 min at 14.000 rpm (Eppendorf Centrifuge 5415C). 

### 2.4. Fluorescence Anisotropy Measurements

**Figure 3:**
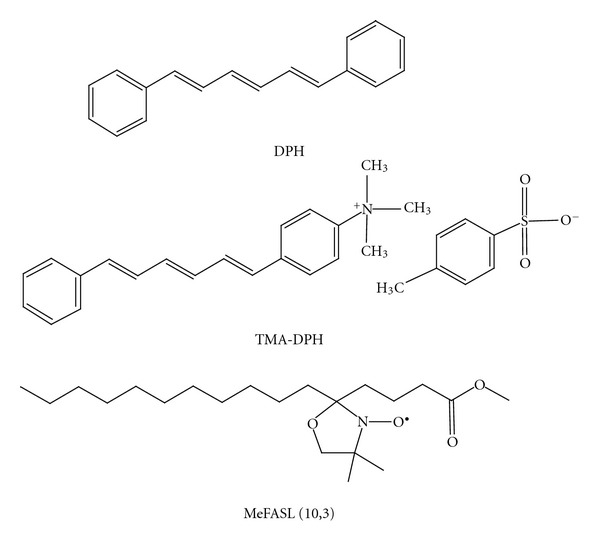
Structural formulas of 1,6-diphenyl-1,3,5-hexatriene (DPH), trimethylammonium-6-phenyl-1,3,5-hexatriene (TMA-DPH), and methylester of 5-doxyl palmitic acid [MeFASL (10,3)].

Fluorescence anisotropy measurements of 1,6-diphenyl-1,3,5-hexatriene (DPH) and trimethylammonium-6-phenyl-1,3,5-hexatriene (TMA-DPH) (Figure 3) in PLMF archaeosomes were performed in a 10 mm-path-length cuvette using a Cary Eclipse fluorescence spectrophotometer (Varian, Mulgrave, Australia), in the temperature range from 20°C to 90°C, and the pH range from 6.0 to 8.0 in the relevant buffer solutions. Varian autopolarizers were used, with slit widths with a nominal band-pass of 5 nm for both excitation and emission. Here, 10 *μ*L DPH or TMA-DPH (Sigma-Aldrich Chemie GmbH, Steinheim, Germany) in dimethyl sulphoxide (Merck KGaA, Darmstadt, Germany) was added to 2.5 mL 100 *μ*M solutions of SUVs prepared from the PLMF from *A. pernix *in the relevant buffer, to reach a final concentration of 0.5 *μ*M DPH and 1.0 *μ*M TMA-DPH. DPH and TMA-DPH fluorescence anisotropy was measured at the excitation wavelength of 358 nm, with the excitation polarizer oriented in the vertical position, while the vertical and horizontal components of the polarized emission light were recorded through a monochromator at 410 nm for both probes. The emission fluorescence of DPH and TMA-DPH in aqueous solution is negligible. The anisotropy (*r*) was calculated using the built-in software of the instrument (1):

(1)r=I||−I⊥I||+2I⊥,

where, *I*_||_ and *I*_⊥_ are the parallel and perpendicular emission intensities, respectively. The values of the G-factor [the ratio of the sensitivities of the detection system for vertically (*I*_*HV*_) and horizontally polarized light (*I*_*HH*_)] were determined for each sample separately.

The lipid-order parameter *S* was calculated from the anisotropy using the analytical expression given in (2) [16]:

(2)S=[1−2(r/r0)+5(r/r0)2]1/2−1+r/r02(r/r0),

where *r*_0_ is the fluorescence anisotropy of DPH in the absence of any rotational motion of the probe. The theoretical value of *r*_0_ of DPH is 0.4, while the experimental values of *r*_0_ lie between 0.362 and 0.394 [16]. In our calculation, the experimental value of *r*_0_ = 0.370 and *r*_0_ = 0.369 for DPH and TMA-DPH in DPPC at 5°C was used, respectively.

### 2.5. Electron Paramagnetic Resonance Measurements

For the EPR measurements, the PLMF SUVs were spin-labelled with a methylester of 5-doxyl palmitic acid [MeFASL(10,3)] (Figure 3), and the EPR spectra recorded with a Bruker ESP 300 X-band spectrometer (Bruker Analytische Messtechnik, Rheinstein, Germany). The MeFASL(10,3) lipophilic probe was selected due to its moderate stability in the membrane and its relatively high-resolution capability for local membrane ordering and dynamics. It is dissolved in the phospholipid bilayer with nitroxide group located in the upper part of the layers. 

With the MeFASL(10,3) film dried on the wall of a glass tube, 50 *μ*L 10 mg*·*mL^−1^ PLMF SUVs in the relevant buffer was added, and the sample was vortexed for 15 min. This was designed for a final molar ratio of MeFASL(10,3): lipids of 1 : 250. The sample was transferred to a capillary (75 mm; Euroglas, Slovenia), and the EPR spectra were recorded using the following parameters: centre field, 332 mT; scan range, 10 mT; microwave power, 20.05 mW; microwave frequency, 9.32 GHz; modulation frequency, 100 kHz; modulation amplitude, 0.2 mT; temperature range; 5°C to 95°C. Each spectrum was the average of 10 scans, to improve the signal-to-noise ratio. From the EPR spectra, the mean empirical correlation time (*τ*_*c*_) was calculated using (3) [17]:

(3)τc=kΔH0[(h0/h−1)1/2−1].

The line width (*Δ*H*_0_*; in mT) and the heights of the mid-field (*h*_0_)and high-field (*h*_−1_) lines were obtained from the EPR spectrum (Figure 6); *k* is a constant typical for the spin probe, which is 5.9387 × 10^−11^ mT^−1^ for MeFASL (10,3) [17]. 

### 2.6. Computer Simulation of the EPR Spectra

For more precise descriptions of the membrane characteristics, computer simulations of the EPR spectra line shapes were performed using the EPRSIM programme (Janez Štrancar, 1996-2003, http://www2.ijs.si/~jstrancar/software.htm). Generally, to describe the EPR spectra of spin labels, the stochastic Liouville equation is used [18–20]. However, in a membrane system labelled with fatty acid spin probes, local rotational motions are fast with respect to the EPR time scale. Modeling of the spectra taken at physiological temperature is therefore simplified by restricting the motions to the fast motional regime. Since the basic approach was already discussed elsewhere [21, 22], it is only summarized here. The model takes into account that the membrane is heterogeneous, and is composed of several regions that have different fluidity characteristics. Therefore, the EPR spectra are composed of several spectral components that reflect the different modes of restricted rotational motion of the spin probe molecules in the different membrane environments. Each spectral component is described by a set of spectral parameters that define the line shape. These are the order parameter (*S*), the rotational correlation time (*τ*_*c*_), the line width correction (*W*), and the polarity correction factors of the magnetic tensors *g* and *A* (*p*_*g*_ and *p*_*A*_, resp.). The *S* describes the orientational order of the phospholipid alkyl chains in the membrane domains, with *S* = 1 for perfectly ordered chains and *S* = 0 for isotropic alignment of the chains. Membrane domains that are more fluid are characterized by a smaller *S*. The *τ*_*c*_ describes the dynamics of the alky chain motion, with the *W* due to the unresolved hydrogen superhyperfine interactions, and contributions from other paramagnetic impurities (e.g., oxygen, external magnetic field inhomogeneities). The *p*_*g*_ and *p*_*A*_ polarity correction factors arise from the polarity of the environment of the spin probe nitroxide group (*p*_*g*_ and *p*_*A*_ are large in more polar environment and are below 1 in hydrophobic region). Beside these parameters, the line shape of the EPR spectra is defined by the relative proportions of each of the spectral components (*d*), which describes the relative amount of the spin probe with a particular motional mode, and which depends on the distribution of the spin probe between the coexisting domains with different fluidity characteristics. As the partition of the MeFASL (10,3) was found to be approximately equal between the different types of domains of phospholipid/cholesterol vesicles [23], we assumed that the same is valid also for these PLMF liposomes.

It should be stressed that the lateral motion of the spin probe is slow on the time scale of the EPR spectra [24]. Therefore, an EPR spectrum describes only the properties of the nearest surroundings of a spin probe on the nm scale. All of the regions in the membrane with similar modes of spin probe motion contribute to one and the same spectral component. Thus, each spectral component reflects the fluidity characteristics of a certain type of membrane nanodomain (with dimensions of several nm) [25].

To obtain best fit of calculated-to-experimental spectra, stochastic and population-based genetic algorithm is combined with Simplex Downhill optimization method into the evolutionary optimization method (HEO), which requires no special starting points and no user intervention [26]. In order to get a reasonable characterization one still has to define the number of spectral components before applying the optimization. To resolve this problem multirun HEO optimization is used together with a newly developed GHOST condensation procedure. According to this method, 200 independent HEO simulation runs for each EPR spectrum were applied, taking into account 4 different motional modes of spin probe (23 spectral parameters), which is around the resolution limit of EPR nitroxide experiments. From these runs only the set of parameters, which correspond to the best fits were used. All the best-fit sets of parameters obtained by 200 optimizations were evaluated according to the goodness of the fit (*χ* ^2^ filter) and according to the similarity of the parameter values of best fits (density filter). The parameters of the best fits were presented by three two-dimensional cross-section plots using four spectral parameters: order parameter *S*, rotational correlation time *τ*_*c*_, line broadening *W*, and polarity correction factor *p*_*A*_ (*S*-*τ*_*c*_, *S*-*W*, and *S*-*p*_*A*_). Groups of solutions, which represent the motional modes of spin probes in particular surrounding and which could correspond to different membrane regions, can be resolved either graphically on GHOST diagrams or numerically within GHOST condensation. Starting values of parameters of spectral components were defined using the average parameters taken from the GHOST diagrams [27]. From these plots information about the motional patterns, defined with *S*, *τ*_*c*_, *W*, and *p*_*A*_ in different membrane regions can be obtained. In this way, the changes in the spin probe motional patterns in different membrane regions, due to the interaction of membrane with biologically active compound, due to temperature, pH, and changes in membrane composition, can be studied.

## 3. Results and Discussion

### 3.1. Fluorescence Anisotropy Measurements

Fluorescence probes have been widely used in the study of the structure and dynamic of biological membranes [28]. Their photophysical properties are affected by the physicochemical changes of the microenvironment where the probes are located. Two common probes for the study of membrane properties are DPH and its cationic derivative TMA-DPH. Since DPH is a hydrophobic probe, it is incorporated in the inner apolar core at different positions along the membrane, while the polar group region of TMA-DPH remains anchored at the lipid-water interface of the membrane with the hydrocarbon chain entering the lipid part of the membrane. Xu and London [29] showed that anisotropy values are highest in gel states, lowest in liquid-disordered states, and intermediate in liquid-ordered states. DPH and TMA-DPH *r* depend on the degree of molecular packing of membrane chains and can be related to the order parameter *S*. The fluidity may be defined as the reciprocal of the lipid order parameter *S* [30]. 

**Figure 4:**
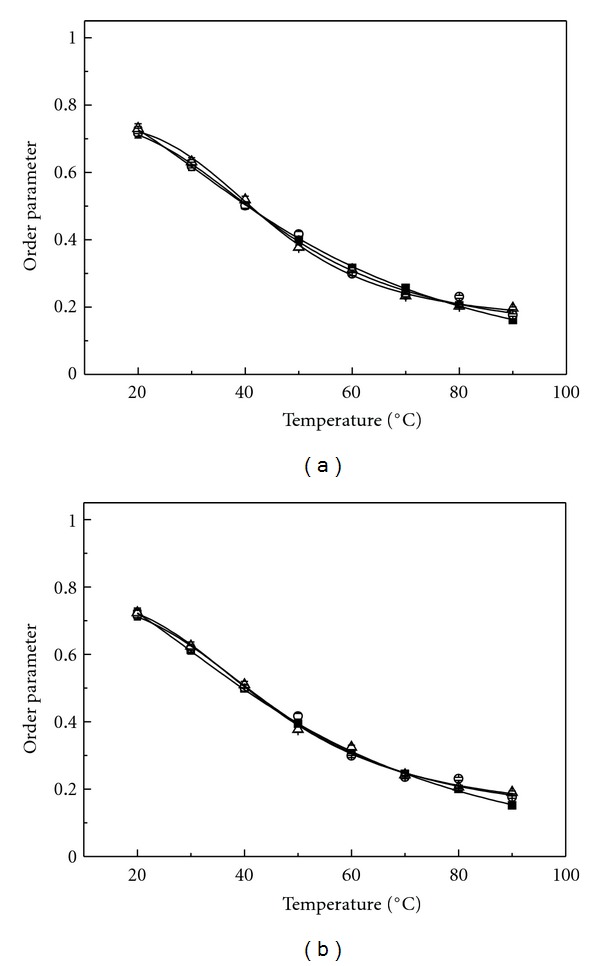
Temperature dependence of the lipid-order parameter of the PLMF from *A. pernix* grown in medium with different pH (■ pH 6.0; ○ pH 7.0; Δ pH 8.0) determined by measuring the anisotropy of DPH. The lines represent nonlinear curve fitting to the data points. (a) pH of measured samples was the same as the pH of growth medium; (b) experiments were performed at pH 7.0.

**Figure 5:**
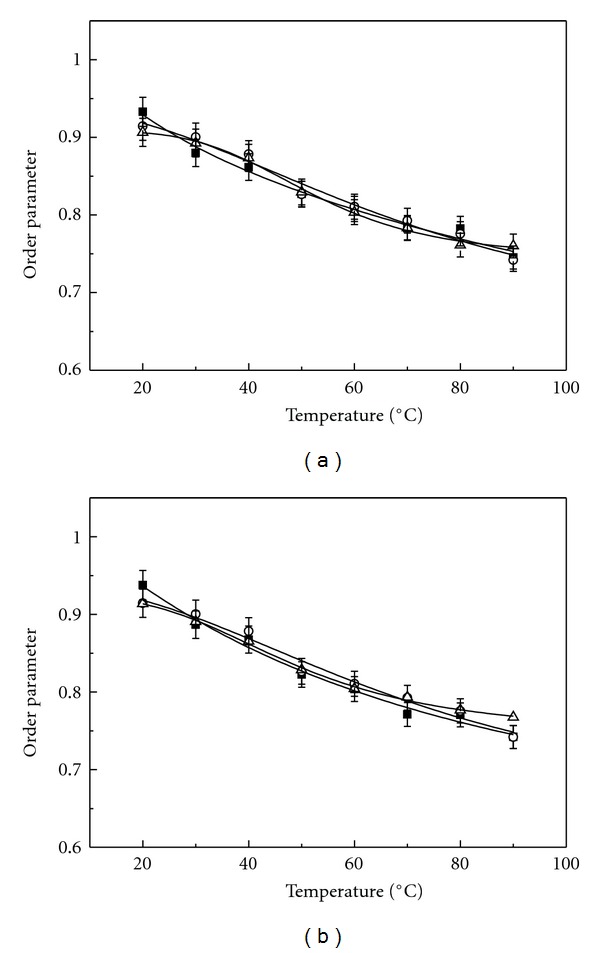
Temperature dependence of the lipid order parameter of the PLMF from *A. pernix* grown in medium with different pH (■ pH 6.0; ○ pH 7.0; Δ pH 8.0) determined by measuring the anisotropy of TMA-DPH. The lines represent nonlinear curve fitting to the data points. (a) pH of measured samples was the same as the pH of growth medium; (b) experiments were performed at pH 7.0.

The levels of order in the SUVs composed of PLMF isolated from *A. pernix* grown at pH 6.0, pH 7.0, and pH 8.0 and measured at the same pHs or at pH 7.0 were calculated from anisotropy measurements of DPH (Figures 4(a) and 4(b)) and TMA-DPH (Figures 5(a) and 5(b)), respectively. No significant differences in the order parameters of archaeosomes were observed regardless the growth medium of the *A. pernix* cells or measured pH in the tested temperature range. The order parameter determined by applying DPH of these archaeosomes steadily decreased with increasing temperature, which indicates a gradual increase in membrane fluidity (Figures 4(a) and 4(b)). Previously, we have shown also by applying DSC that in the range from 0°C to 100°C, the archaeosomes do not undergo gel-to-liquid crystalline phase transition [11]. The initial values of the order parameter of DPH at 20°C were: pH 6.0, 0.72 ± 0.1; pH 7.0, 0.72 ± 0.1; pH 8.0, 0.73 ± 0.1. Similarly, we have not detected the significant differences in the order parameter determined by applying TMA-DPH in archaeosomes regardless the growth or measured pH values. The initial value of order parameter of TMA-DPH in comparison to DPH in archaeal lipids at the same temperature and pH was higher: pH 6.0, 0.93 ± 0.1; pH 7.0, 0.91 ± 0.1; pH 8.0, 0.91 ± 0.1. Another observation, which should be stressed is that the changing in the order parameter determined by TMA-DPH is less temperature sensitive (Figures 5(a) and 5(b)). This might not be surprising since TMA-DPH is cationic probe located at the lipid-water interface of the membrane and the archaeosomes (SUV) have zeta potential of −50 mV [11]. The zeta potential of archaeosomes (LUV) was not changed with pH in the pH range from 5.0 to 10.0 [11]. It is likely that in the studied pH range from 6.0 to 8.0 the zeta potential of SUV archaeosomes is also not changing, which correlate with no observed changes in TMA-DPH anisotropy with pH.

 The fact that we have not determined any significant differences in the behaviour of two fluorescence probes regardless the pH of growth medium of *A. pernix*, suggest that the lipid composition is not changing in the studied pH range of growing (from pH 6.0 to 8.0) or in the measured pH range from 6.0 to 8.0. This statement was supported by the TLC results of PLMF of *A. pernix *growth at different pHs (Figure 2). The ratio between two major lipids component in *A. pernix* membrane C_25,25_-archetidylinositol (AI) and C_25,25_-archetidyl(glucosyl)inositol (AGI) is at growth pH 6.0 and 7.0: 9 ± 1% of AI and 91 ± 1% of AGI and at growth pH 8.0: 8 ± 1% of AI and 92 ± 1% of AGI.

### 3.2. Electron Paramagnetic Resonance Measurements

**Figure 6:**
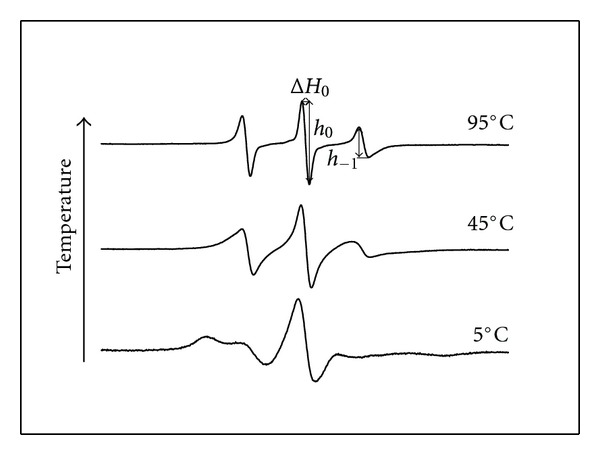
Representative EPR spectra of MeFASL(10,3) in the membrane of the SUV archaeosomes at pH 7.0 prepared from the PLMF isolated from *A. pernix* grown at pH 7.0.

**Figure 7:**
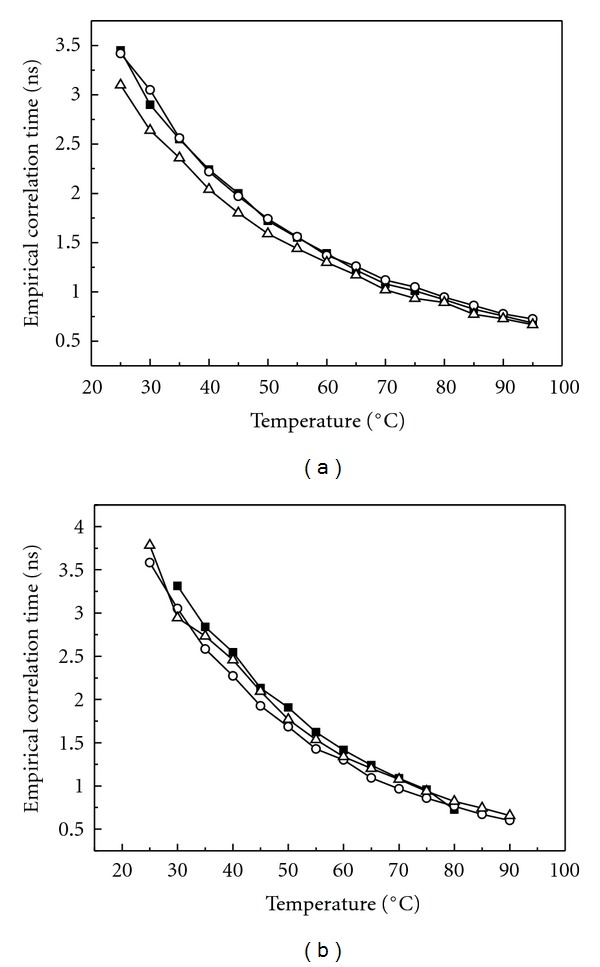
Temperature dependence of empirical correlation time (*τ*_*c*_) of MeFASL(10,3) in SUV archaeosomes prepared from PLMF lipids isolated from *A. pernix* grown in medium with different pH (■ pH 6.0; ○ pH 7.0; Δ pH 8.0) and measured at the same pHs (a) and at pH 7.0 (b). Empirical correlation time was calculated directly from the EPR spectra according to Equation (3).

The empirical correlation times of MeFASL(10,3) in these liposomes prepared from the PLMF isolated from *A. pernix* were measured directly from the EPR spectra (Figure 6). These decreased gradually with temperature and did not show significant differences with respect to the pH of the growth medium (Figure 7). The empirical correlation time reflect an average ordering and dynamics of the phospholipid alkyl chains in the spin-probe nitroxide group surrounding and is in inverse relation to membrane fluidity. The data correlate well with fluorescence anisotropy measurements of DPH incorporated into archaeosomes, which shows that the membrane fluidity increases with temperature, but on average it does not depend on the pH of the growth medium. Similar results have been reported for archaeosomes composed of bipolar tetraether lipids [31].

**Figure 8:**
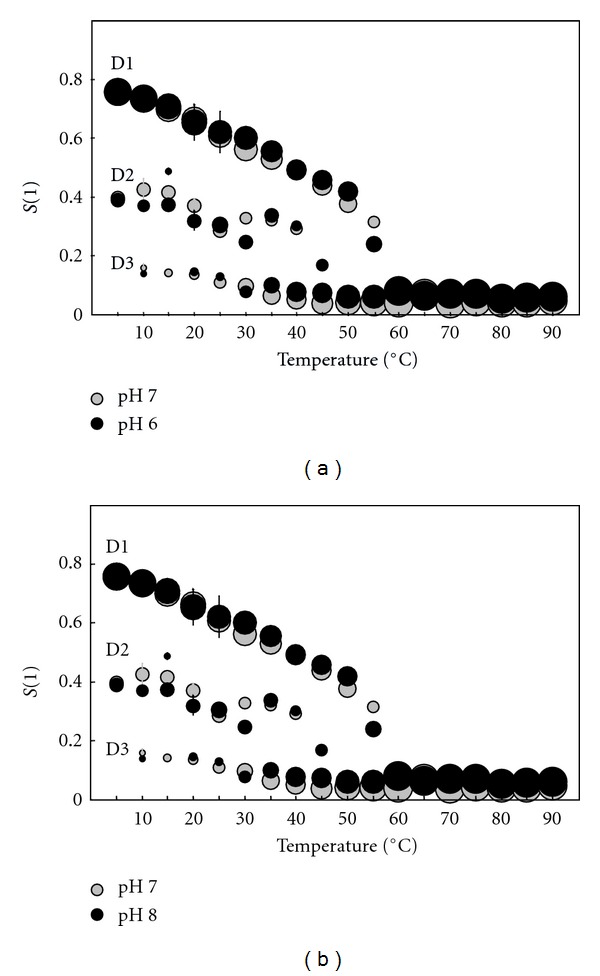
Temperature dependence of the order parameters (*S*) and proportions of MeFASL(10,3) in the membrane regions of the SUV archaeosomes prepared from the PLMF from *A. pernix* grown at pH 6.0 (black circles) and 7.0 (grey circles) (a), and at pH 7.0 (grey circles) and 8.0 (black circles) (b). The diameters of the symbols indicate the proportions of each region. D1, D2, and D3 indicate the regions with the highest, intermediate-and lowest-order parameters, respectively. Experiments were performed at pH 7.0.

To obtain more detailed information about the possible influences of different growth medium pH on the membrane structural characteristics and on their changes with temperature, computer simulations of the EPR spectra were performed. At temperatures below 70°C, good fits with the experimental spectra were obtained taking into account that the spectra are composed of at least three spectral components. This indicates that the archaeosome membranes are heterogeneous and composed of several regions with different modes of spin-probe motions. All of the regions in the membranes with the same fluidity characteristics are described by a single spectral component. The corresponding EPR parameters determine motional pattern of the spin probe, irrespective to its location in the membrane. Smaller regions with the same physical characteristics could not be distinguished from few large regions. This also means that EPR does not necessarily reflect directly the macroscopic properties of the membrane or large membrane domains, but reflects also the membrane superstructure on nm scale. The three motional patterns of spin probe observed could be due to the two-component lipid composition (AI and AGI) of the membrane. Additionally, some dynamic fluctuations of phospholipids or vertical motion of spin probe within the membrane can be detected as a specific motional pattern of spin probe. These motional patterns could be altered if the membrane is influenced by some external perturbations or if the membrane composition is changed. At higher temperatures, the membranes become more homogeneous and can be described by only one spectral component. The changes in the order parameters of the different membrane regions and their proportions with temperature are shown in the form of bubble diagrams in Figure 8, where the dimensions of each symbol represent the proportions of the spin probes in the corresponding membrane regions. With increasing temperature, the order parameter of the most ordered region decreases, its proportion decreases and disappears in the temperature region between 55°C and 65°C. The proportions of the less ordered regions increase with increasing temperature, and above 70°C these remain unchanged. The calculated order parameters for the samples grown at different pH and measured at pH 7.0 are in the range uncertainty of the calculation. 

 Order parameters obtained by fluorescence measurement (Figures 4 and 5) and those obtained by computer simulation of EPR spectra (Figure 8) cannot be directly compared since the three probes (Figure 3), which differ appreciably in their shape and dimensions cause different perturbations in their surrounding and monitors the properties at different depth of the membrane. DPH is highly hydrophobic and reflects the properties in the inner apolar core at different positions along the membrane, TMA-DPH is anchored at water-lipid interface, while MeFASL(10,3) with nitroxide group on the 5th C atom (counting from the methyl-ester group) monitors the properties in the upper part of phospholipid layers but exhibit also some translational motion within the membrane. Besides by fluorescence polarization measurements an average order parameter in the membrane is obtained, while by computer simulation of EPR spectra order parameter is distinguished from the rotational rate and reflects differe surroundings of the spin probe at lower temperatures, which could be due to membrane heterogeneity produced by distribution between AI and AGI of the membrane but can as well be the consequence of some fluctuations or vertical motion of the spin probe within the bilayer, which seems to be influenced by temperature. 

## 4. Conclusions

Fluorescence anisotropy measurements of DPH and TMA-DPH in addition to EPR spectrometry here showed steady decreases in the order parameter of archaeal lipids with increasing temperature, regardless the pH of growth of archaea or measuring pH. This indicates a gradual increase in the membrane fluidity in all of these samples, although no significant differences were seen for the influence of the *A. pernix* growth medium pH. TMA-DPH located close to water-lipid interface shows less temperature dependence in comparison of DPH or MeFASL(10,3). The more detailed analysis using computer simulation of the EPR spectra revealed membrane heterogeneity at temperatures below 55°C, which disappears at higher temperatures. But the EPR parameters calculated from the spectra of archaeosomes obtained from the PLMF from *A. pernix* grown at different pH and measured at pH 7.0 remains in the range of the calculation uncertainty. The results are supported by TLC analysis of isolated lipids, which show no significant differences between PLMF isolated from *A. pernix* grown in medium with different pHs.

To summarize, the present data showed that cell growth pH has no effect on membrane properties being examined. The previous *in vivo* study [14] showed that the cell growth varies with medium pH. This discrepancy is interesting since the polar lipids of *A. pernix* K1 consist solely of C_25,25_-archeaol, which has not been changed by growth pH according to our data presented here. Previously, we have reported that the maximum cell density of *A. pernix* growth at pH 7.0 and 8.0 conditions were similar, while a significantly lower maximum cell density was obtained at pH 6.0 and no growth at pH 5.0 [14]. It is likely that at pHs lower than 6.0 the membranes of the neutrophilic *A. pernix* composed of C_25,25_-archaeol becomes proton permeable and that the permeability is not regulated by lipid composition.
